# Utilizing deep learning-based causal inference to explore vancomycin’s impact on continuous kidney replacement therapy necessity in blood culture-positive intensive care unit patients

**DOI:** 10.1128/spectrum.02662-24

**Published:** 2024-12-10

**Authors:** Min Woo Kang, Yoonjin Kang

**Affiliations:** 1 Department of Internal Medicine, Seoul National University Hospital, Seoul, South Korea; 2 Department of Thoracic and Cardiovascular Surgery, Seoul National University Hospital, Seoul National University, College of Medicine, Seoul, South Korea; University of Guelph College of Biological Science, Guelph, Ontario, Canada

**Keywords:** blood culture positive, continuous kidney replacement therapy, vancomycin, machine learning, deep learning, causal inference

## Abstract

Patients with positive blood cultures in the intensive care unit (ICU) are at high risk for septic acute kidney injury requiring continuous kidney replacement therapy (CKRT), especially when treated with vancomycin. This study developed a machine learning model to predict CKRT and examined vancomycin’s impact using deep learning-based causal inference. We analyzed ICU patients with positive blood cultures, utilizing the Medical Information Mart for Intensive Care III data set. The primary outcome was defined as the initiation of CKRT during the ICU stay. The machine learning models were developed to predict the outcome. The deep learning-based causal inference model was utilized to quantitatively demonstrate the impact of vancomycin on the probability of CKRT initiation. Logistic regression was performed to analyze the relationship between the variables and the susceptibility of vancomycin. A total of 1,318 patients were included in the analysis, with 41 requiring CKRT. The Random Forest and Light Gradient Boosting Machine exhibited the best performance, with Area Under Curve of Receiver Operating Characteristic Curve values of 0.905 and 0.886, respectively. The deep learning-based causal inference model demonstrated an average 7.7% increase in the probability of CKRT occurrence when administrating vancomycin in total data set. Additionally, that younger age, lower diastolic blood pressure, higher heart rate, higher baseline creatinine, and lower bicarbonate levels sensitized the probability of CKRT application in response to vancomycin treatment. Deep learning-based causal inference models showed that vancomycin administration increases CKRT risk, identifying specific patient characteristics associated with higher susceptibility.

**IMPORTANCE:**

This study assesses the impact of vancomycin on the risk of continuous kidney replacement therapy (CKRT) in intensive care unit (ICU) patients with blood culture-positive infections. Utilizing deep learning-based causal inference and machine learning models, the research quantifies how vancomycin administration increases CKRT risk by an average of 7.7%. Key variables influencing susceptibility include baseline creatinine, diastolic blood pressure, heart rate, and bicarbonate levels. These findings offer insights into managing vancomycin-induced kidney risk and may inform patient-specific treatment strategies in ICU settings.

## INTRODUCTION

In the realm of critical care medicine, blood culture positive, including bacteremia and fungemia, represents a significant concern due to its potential to progress to sepsis—a condition necessitating close monitoring or invasive treatment within intensive care units (ICUs). Sepsis is notorious for its ability to induce multi-organ failure, with acute kidney injury (AKI) occurring in a significant percentage of cases (1–3). Notably, a considerable portion of patients with septic AKI reach a severity necessitating kidney replacement therapy, with many of these cases being hemodynamically unstable, thereby requiring continuous kidney replacement therapy (CKRT) for management (4).

Beyond the direct impact of sepsis-induced AKI, the nephrotoxicity of antibiotics administered poses an additional risk for the development of AKI. Among these antibiotics, vancomycin is well-recognized for its nephrotoxic potential, occasionally leading to kidney replacement therapy requiring AKI (5, 6). The administration of vancomycin in patients with sepsis is thus associated with an elevated risk of AKI. However, despite these risks associated with vancomycin, there are instances where it is essential for treating sepsis patients, and it is often administered empirically.

This research endeavored to develop a predictive model for identifying blood culture-positive patients at the time of ICU admission who are likely to require CKRT using machine learning algorithms, using Medical Information Mart for Intensive Care III (MIMIC-III) database. Considering the need to evaluate vancomycin’s dual impact—septic AKI reduction from infection control and AKI risk from nephrotoxicity—we limited our analysis to patients with confirmed septicemia through positive blood culture results. Furthermore, utilizing a deep learning-based causal inference model, this study aimed to quantitatively analyze the impact of vancomycin administration on the occurrence of CKRT-necessitating severe kidney function impairment. A particular focus was on identifying patient subgroups at heightened risk, thereby providing insights that could guide more nuanced clinical decision-making in the management of sepsis when administration of vancomycin.

## MATERIALS AND METHODS

### Study population and data source

This study utilized the MIMIC-III database, a large, freely available database comprising de-identified health-related data associated with over 40,000 patients who stayed in critical care units of the Beth Israel Deaconess Medical Center between 2001 and 2012 (7). MIMIC-III integrated clinical data from various sources, including laboratory results, electronic health records, and treatment information. Our study cohort comprised patients admitted to the ICU with positive blood culture tests conducted within a 48-hour window prior to ICU admission and up to 6 hours after admission.

### Variables for analysis

Vancomycin administration was defined based on the medication administration records within MIMIC-III, identifying patients who received vancomycin surrounding their ICU admission. The primary outcome, CKRT application, was defined as at least one instance of CKRT during the ICU stay. Demographic data (age and sex) and initial vital signs (systolic blood pressure [SBP], diastolic blood pressure [DBP], heart rate, and oxygen saturation [SpO_2_]) were collected at the time of ICU admission. Comprehensive laboratory data (white blood cell count [WBC], hemoglobin, platelets, prothrombin time international normalized ratio [PT INR], activated partial thromboplastin time [aPTT], creatinine, pH, anion gap, bicarbonate, sodium, and potassium) were taken from measurements closest to the time of ICU admission, ranging from 24 hours before to 6 hours after admission. The baseline creatinine was defined as the lowest recorded creatinine value within 3 months prior to ICU admission and was utilized for analysis. Additionally, specific blood culture-positive results (Methicillin-susceptible *Staphylococcus aureus* [MSSA], Methicillin-resistant *Staphylococcus aureus* [MRSA], *Pseudomonas*, and *Candidemia*), the rate of norepinephrine administration, and the occurrence of intubation within the initial 6 hours after ICU admission were analyzed. For analyzing differences between the groups with and without CKRT applying, continuous variables were compared using *t*-tests, while categorical variables utilized *χ*^2^ tests.

### Logistic regression and machine learning models predicting CKRT applying

In this study, a multivariable logistic regression analysis was initially employed to assess the relationship between conducting CKRT and variables, especially vancomycin administration, with statistical significance set at a *P* value < 0.05. Following this, a comprehensive machine learning analysis was conducted using the Python package PyCaret, engaging a diverse array of algorithms such as Extra Trees, CatBoost, Extreme Gradient Boosting, Random Forest (RF), Light Gradient Boosting Machine (LGBM), Gradient Boosting, and Decision Tree. Through 10-fold cross-validation on the train data set, the two models exhibiting the highest area under the receiver operating characteristic (AUROC) curve were identified for further exploration. After hyperparameter tuning with 10-fold cross-validation, the best-performing models based on AUROC were rigorously tested for accuracy and AUROC on the test data set, and the importance of variables in predicting the application of CKRT was analyzed. To deepen the analysis, SHAP (SHapley Additive exPlanations) values were calculated for the total data set using the top two algorithms, allowing for the creation of Beeswarm plots that illustrate the impact of each variable on CKRT application (8). SHAP values, based on game theory, demystify machine learning prediction process by quantifying the contribution of each input variable. This method enhances model transparency and assists in pinpointing critical factors affecting CKRT use.

### Deep learning-based causal inference model

Similar to the approach taken in the machine learning analysis, the data set was partitioned into train and test data sets for the causal inference analysis. The Generative Adversarial Nets for Inference in Treatment Effects (GANITE) model was employed, with vancomycin administration serving as the treatment variable and the application of CKRT as the outcome, to train the model (9). The efficacy of the model was evaluated on the test data set, focusing on metrics such as accuracy and the AUROC. Additionally, the average treatment effect (ATE) of vancomycin on CKRT application was assessed across the train, test, and entire data sets, aiming to determine whether the administration of vancomycin, on average, led to an increase or decrease in the application of CKRT probability. The trained model was also applied to two specific patients within the data set to calculate the individual treatment effect, demonstrating how the model can be used to assess vancomycin susceptibility for individual patients. Moreover, to delve deeper into the impact of vancomycin on CKRT application, the data set was split into two groups based on the extent to which vancomycin administration influenced the likelihood of CKRT application: one group where the influence of vancomycin on increasing the probability of CKRT application was greater than the median value, and another group where the influence was less than or equal to the median. The differences in continuous variables between these groups were analyzed using *t*-tests, while categorical variables were compared using *χ*^2^ tests. Furthermore, a multivariable logistic regression analysis was conducted to examine the odds ratio (OR) for the treatment effect of vancomycin administration exceeding the median value, thereby identifying which variable characteristics exert a more pronounced effect on the probability of CKRT application following vancomycin administration, indicating susceptibility to vancomycin.

## RESULTS

### Baseline characteristics of the study population

**Fig 1:**
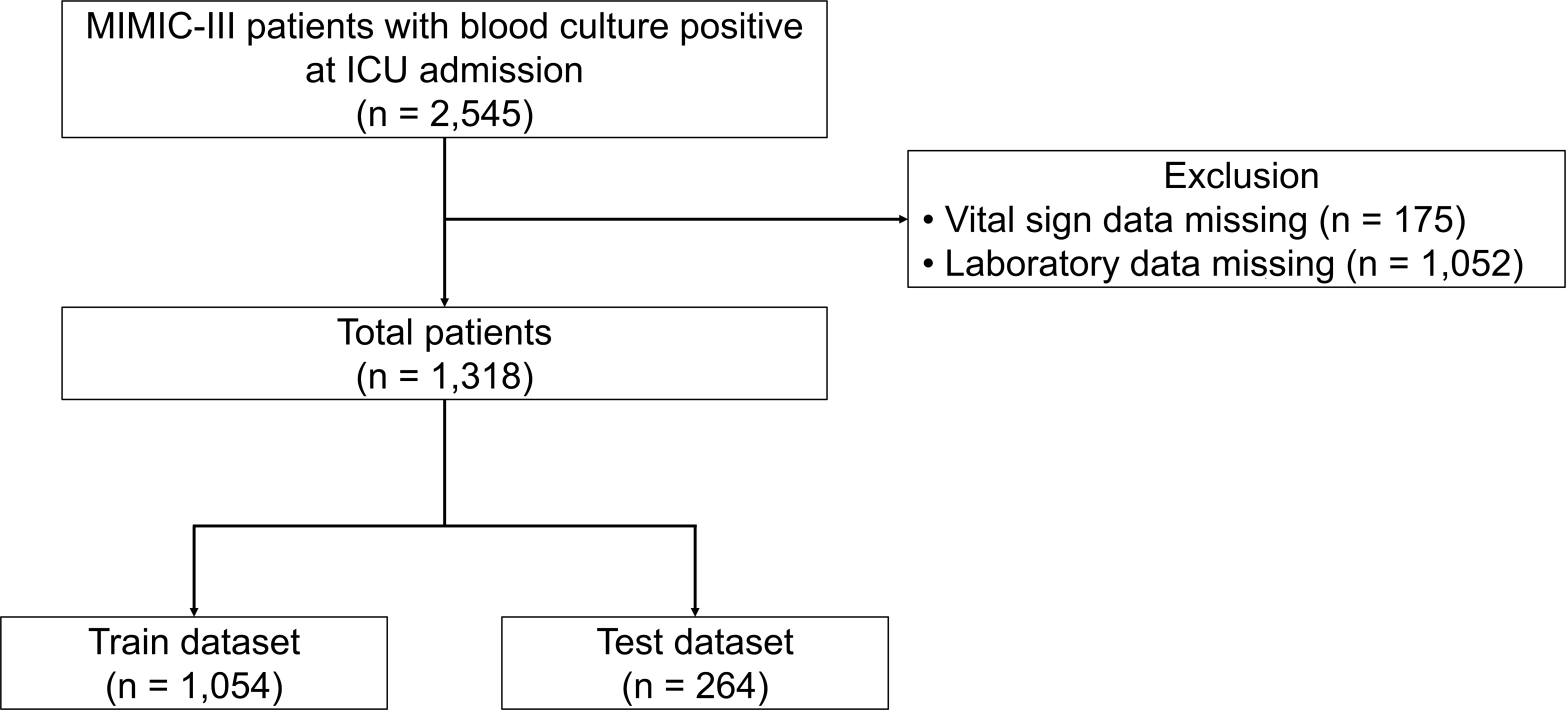
Flow diagram of the study population.

The study encompassed 1,318 patients, with 41 (3.1%) requiring CKRT during their ICU stay (Fig. 1; Table 1). The average age of the study population was 64.9 years, with a male predominance of 58.0%. CKRT recipients were notably younger, averaging 57.6 years. Patients requiring CKRT had markedly elevated initial creatinine levels (3.55 vs 2.07 mg/dL, *P*: 0.007) and baseline creatinine (2.94 vs 1.84 mg/dL, *P*: 0.013). Among the patients, 31.7% who underwent CKRT received vancomycin, compared to only 11.2% of those who did not undergo CKRT, a difference that was statistically significant (*P*: <0.001). Subsequently, the total data set was partitioned into train data set (1,054 patients) and test data set (264 patients), with no significant differences observed in most variables between the two groups (Table S1).

**TABLE 1:** Baseline characteristics

	Total (*N* = 1,318)	CKRT (*N* = 41)	No CKRT (*N* = 1,277)	*P*-value^*a*^
Age (years)	64.88 ± 15.0	57.63 ± 11.46	65.11 ± 15.05	<0.001
Male (%)	764 (58.0)	21 (51.2)	743 (58.2)	0.466
SBP (mmHg)	115.0 ± 25.36	108.32 ± 25.09	115.22 ± 25.33	0.094
DBP (mmHg)	64.19 ± 143.17	56.39 ± 17.36	64.44 ± 145.41	0.102
Heart rate (/min)	100.12 ± 21.55	102.95 ± 20.21	100.03 ± 21.59	0.375
SpO_2_ (%)	96.12 ± 6.66	96.07 ± 4.21	96.12 ± 6.73	0.949
Creatinine (mg/dL)	2.12 ± 2.1	3.55 ± 3.28	2.07 ± 2.03	0.007
Baseline creatinine (mg/dL)	1.88 ± 1.93	2.94 ± 2.67	1.84 ± 1.89	0.013
Intubation (%)	118 (9.0)	20 (48.8)	98 (7.7)	<0.001
Pseudomonas (%)	25 (1.9)	0 (0.0)	25 (2.0)	<0.001
Candidemia (%)	39 (3.0)	4 (9.8)	35 (2.7)	0.032
MRSA (%)	132 (10.0)	4 (9.8)	128 (10.0)	>0.999
MSSA (%)	99 (7.5)	2 (4.9)	97 (7.6)	0.727
pH	7.35 ± 0.12	7.3 ± 0.15	7.35 ± 0.11	0.044
Anion gap (mmol/L)	16.79 ± 5.17	19.56 ± 6.5	16.7 ± 5.09	0.009
Bicarbonate (mmol/L)	21.81 ± 6.34	19.39 ± 6.22	21.88 ± 6.33	0.016
WBC (10³/μL)	13.89 ± 11.44	13.46 ± 9.89	13.9 ± 11.49	0.783
Hemoglobin (g/dL)	10.56 ± 2.11	9.77 ± 1.96	10.58 ± 2.11	0.014
Platelet (10³/μL)	223.34 ± 137.59	133.83 ± 98.25	226.22 ± 137.71	<0.001
aPTT (sec)	40.1 ± 24.34	50.1 ± 33.71	39.78 ± 23.91	0.062
PT INR	1.77 ± 1.38	2.09 ± 1.38	1.76 ± 1.38	0.151
Sodium (mmol/L)	138.08 ± 5.86	137.93 ± 5.74	138.09 ± 5.87	0.864
Potassium (mmol/L)	4.19 ± 0.82	4.46 ± 0.85	4.18 ± 0.82	0.047
Norepinephrine (mcg/kg/min)	0.19 ± 0.97	0.87 ± 2.19	0.17 ± 0.9	0.052
Vancomycin (%)	156 (11.8)	13 (31.7)	143 (11.2)	<0.001

^*a*^ *χ*^2^ test for categorical variables and *t*-test for continuous variables.

### Multivariable logistic regression for CKRT initiation

After adjusting for confounders, vancomycin administration was significantly associated with CKRT initiation (OR: 2.63 [1.14–6.07], *P*: 0.024; Table S2). Additionally, the initial intubation and younger age were also found to be significantly associated with the application of CKRT.

### Machine learning for predicting CKRT initiation, including variable importance and SHAP value

**Fig 2:**
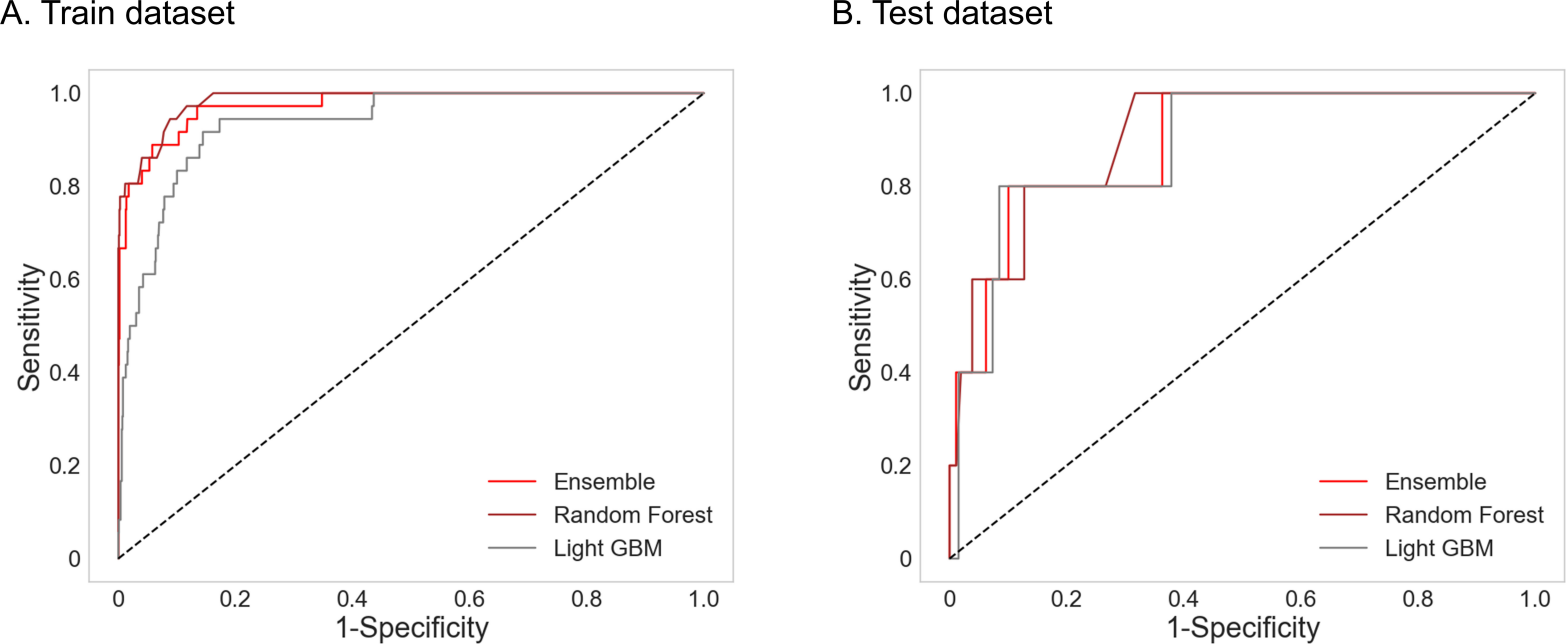
The receiver operating characteristic curves of machine learning prediction models. (A) Train data set. (B) Test data set.

The RF model demonstrated superior performance with an AUROC of 0.905 on the test data set, while the LGBM achieved an AUROC of 0.886, marking these two as the highest-performing machine learning models in our analysis (Fig. 2; Table 2). Conversely, the TabTransformer model exhibited suboptimal performance with an area under the curve (AUC) of 0.693.

**TABLE 2:** Performance of prediction models

	Train data set AUC	Train data set accuracy	Test data set AUC	Test data set accuracy
Ensemble	0.974	0.979	0.893	0.932
RF	0.984	0.988	0.905	0.951
LGBM	0.935	0.942	0.886	0.905

**Fig 3:**
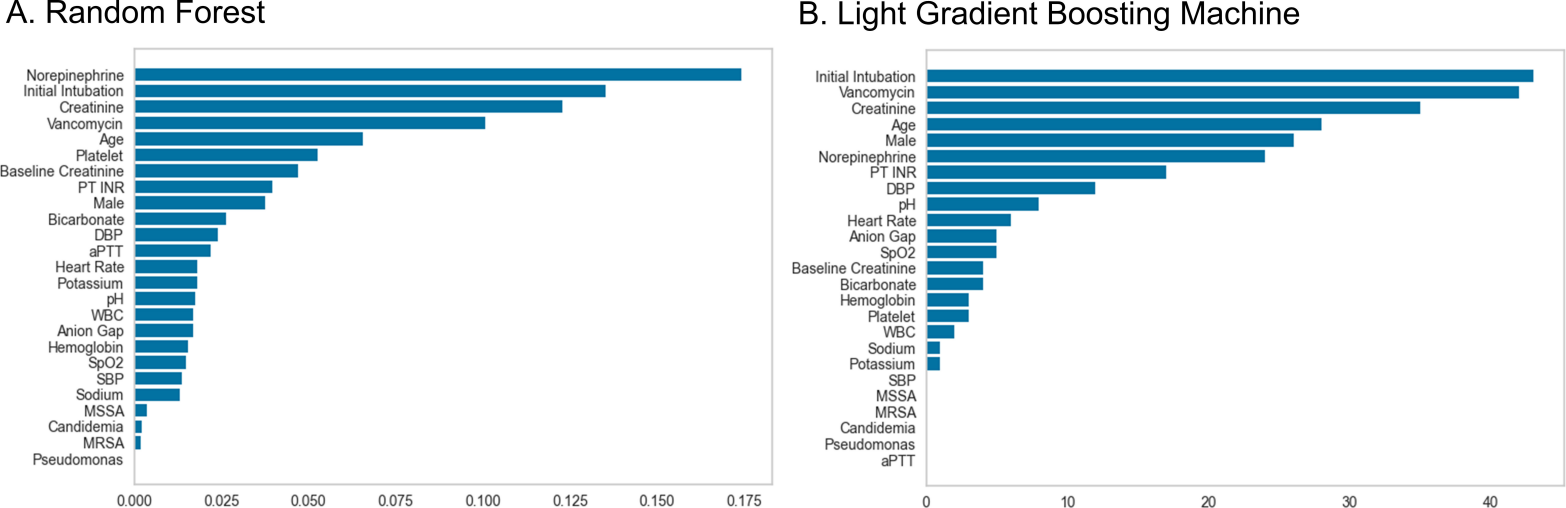
Variable importances of of machine learning prediction models. (A) RF. (B) LGBM.

**Fig 4:**
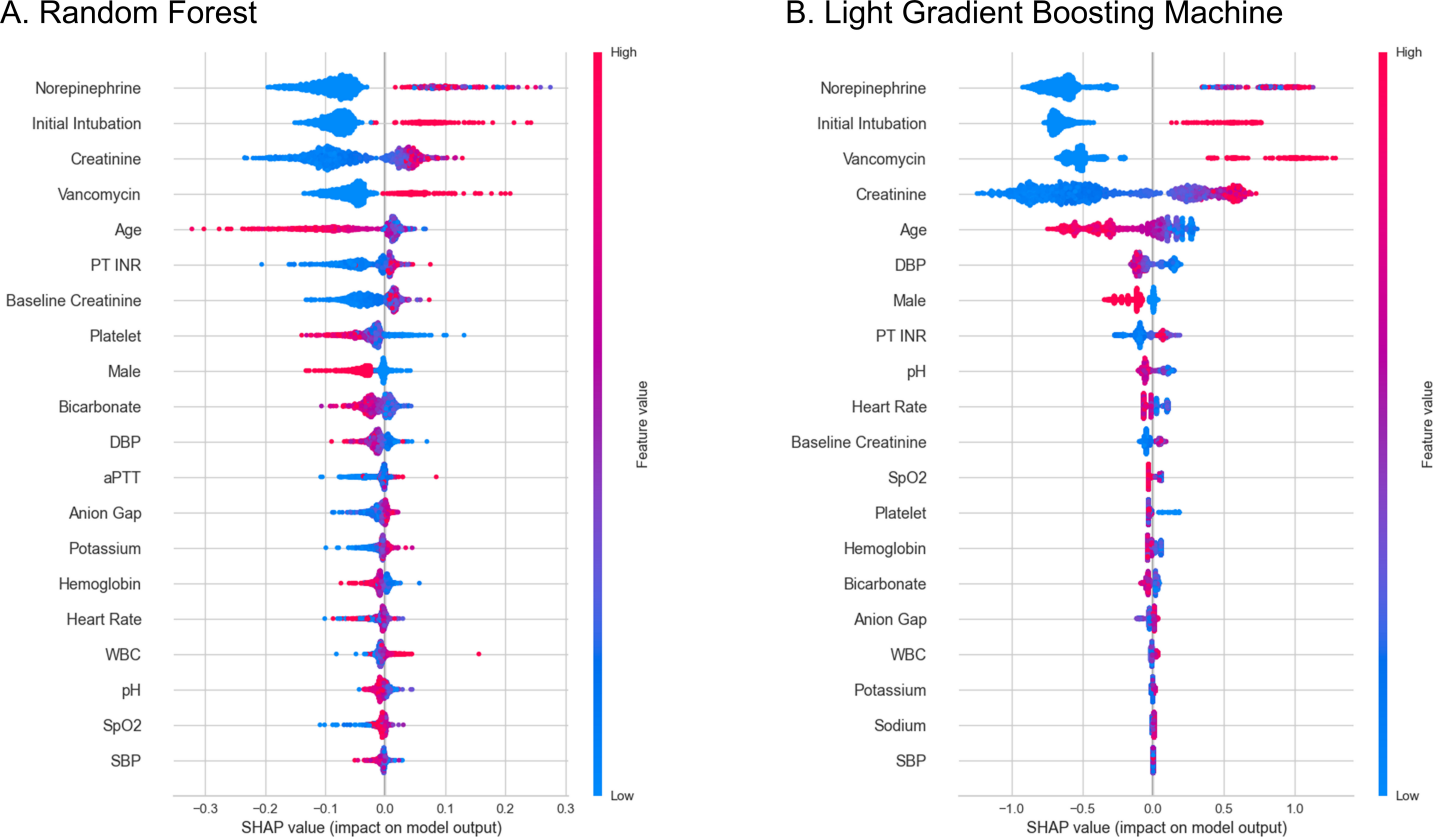
SHAP Beeswarm plot of machine learning prediction models. (A) RF. (B) LGBM.

In variable importance analysis, vancomycin administration was identified as the fourth most important variable in the RF model and the second most important variable in the LGBM model (Fig. 3). Additionally, initial intubation and creatinine levels were highlighted as significant variables in both models. In the SHAP Beeswarm plots for both the RF and LGBM models, the absence of norepinephrine administration, not performing initial intubation, lower levels of creatinine, and not administering vancomycin were highly associated with predictions against the application of CKRT (Fig. 4).

### Deep learning-based causal inference model for identifying the impact of vancomycin

The GANITE model, a deep learning-based causal inference approach, demonstrated that vancomycin administration is associated with an average 7.7% increase in the probability of requiring CKRT in total data set (Table 3). In the test data set, the GANITE model achieved an accuracy of 0.962 and AUROC of 0.786 (Fig. S1). The model was applied to individual patients to provide examples. For the first patient, a 61-year-old with normal vital signs, creatinine levels, and MRSA bacteremia, the probability of requiring CKRT increased by 4.9% when vancomycin was administered. For the second patient, a 68-year-old with low blood pressure, high initial and baseline creatinine levels, and suspected septic shock, the probability of requiring CKRT increased by 11.5% when vancomycin was administered, indicating higher susceptibility (Fig. S2).

**TABLE 3:** Evaluation index and ATE of deep learning-based causal inference model

	Train data set	Test data set	Total data set
Accuracy	0.978	0.962	0.975
AUC	0.917	0.786	0.900
ATE	0.078 (0.074–0.082)	0.074 (0.065–0.083)	0.077 (0.074–0.081)

In the total population, the median value of the effect of vancomycin administration on the application of CKRT was 7.4%. Using this median value as the cut-off value to divide the cohort into two groups, significant differences were observed between the groups in terms of age, SBP, heart rate, SpO_2_, creatinine, baseline creatinine, pH, anion gap, bicarbonate, WBC count, platelets, aPTT, PT INR, and sodium levels in total data set (Table S3). Similar trends were observed in both the train and test data sets (Table S4). A multivariable logistic regression analysis was conducted to assess cases in which vancomycin administration increased the likelihood of applying CKRT by more than 7.4%. The findings indicated that younger age, lower DBP, higher heart rate, lower platelet count, higher aPTT, higher baseline creatinine, and lower bicarbonate levels sensitized the probability of CKRT application in response to vancomycin treatment (Table 4).

**TABLE 4:** OR for high vancomycin susceptibility in CKRT application

	OR (95% CI)	*P*-value
Age	0.96 (0.95–0.97)	<0.001
Male	0.93 (0.69–1.25)	0.631
SBP	1.00 (0.99–1.00)	0.440
DBP	0.97 (0.96–0.98)	<0.001
Heart rate	1.04 (1.03–1.05)	<0.001
SpO_2_	0.99 (0.97–1.01)	0.391
Creatinine	0.84 (0.63–1.11)	0.209
Baseline creatinine	1.56 (1.16–2.09)	0.003
Intubation	0.93 (0.55–1.57)	0.784
Pseudomonas	0.37 (0.12–1.10)	0.073
Candidemia	1.04 (0.41–2.63)	0.940
MRSA	1.39 (0.87–2.23)	0.172
MSSA	1.05 (0.59–1.87)	0.861
pH	3.13 (0.71–13.78)	0.131
Anion gap	1.00 (0.95–1.04)	0.850
Bicarbonate	0.93 (0.90–0.96)	<0.001
WBC	1.00 (0.98–1.01)	0.561
Hemoglobin	1.06 (0.98–1.14)	0.126
Platelet	0.99 (0.99–0.99)	<0.001
aPTT	1.01 (1.00–1.01)	0.042
PT INR	1.06 (0.94–1.19)	0.334
Sodium	1.03 (1.00–1.05)	0.053
Potassium	1.21 (1.00–1.47)	0.050
Norepinephrine	1.03 (0.89–1.20)	0.647

## DISCUSSION

In this investigation, the MIMIC-III ICU database was utilized to develop a machine learning model designed to predict the requirement for CKRT in blood culture-positive patients, with a particular focus on the impact of vancomycin on kidney injury. The insights gained from our study make a substantial contribution to the understanding of the risk of kidney injury among ICU patients with blood culture positive. Previous analyses using MIMIC-III data have extensively explored machine learning, particularly in predicting kidney impairment, and have successfully developed robust models based on prior research (10–12). Our investigation builds upon this knowledge base by specifically addressing the link between vancomycin exposure and CKRT application, thus shedding light on drug-related nephrotoxicity and its predictive indicators, while also utilizing a deep learning-based causal inference model.

Vancomycin administration was associated with the application of CKRT not only in multivariable logistic regression but also in SHAP analysis. The SHAP analysis reveals critical variables that strongly predict the need for CKRT due to significant kidney injury. These variables include the necessity of norepinephrine administration, vancomycin administration, elevated initial creatinine levels, intubation within 6 hours of admission, and younger age. These factors are acknowledged in prior research as risk factors for necessity of CKRT except intubation and younger age (13–16). Patients with respiratory failure severe enough to necessitate intubation are at increased risk for developing AKI and have difficulty compensating acid-base imbalances through respiration (17, 18). Consequently, these conditions could increase the risk of necessitating CKRT. The association between younger age and the application of CKRT may stem from the possibility that older patients often have a higher rate of documented Physician Orders for Life-Sustaining Treatment forms (19), which may lead to a lower likelihood of receiving invasive treatments like CKRT. Consequently, clinicians might opt not to apply CKRT in older patients.

The application of deep learning models in our study marks a significant advancement toward achieving precision medicine in critical care. Research on predicting acute kidney injury in septic patients using similar machine learning approaches has validated their efficacy in clinical forecasting and the identification of key predictors (10–12, 20). Our research extends these methodologies to investigate the causal link between vancomycin and kidney injury requiring CKRT. Consequently, the results of this study indicated that vancomycin causes kidney impairment necessitating CKRT, as evidenced by the causal inference analysis. Moreover, our results revealed that vancomycin administration increases the probability of requiring CKRT, with specific conditions such as low DBP, high heart rate, low platelet count, high baseline creatinine, and low bicarbonate levels further elevating this likelihood. Notably, baseline creatinine levels, rather than creatinine levels at the time of ICU admission, are more sensitive to the increased risk of requiring CKRT following vancomycin administration. Considering these findings, there is a heightened need for caution when administering vancomycin to ICU patients, especially those with identified risk factors.

Furthermore, our deep learning-based causal inference model could refine treatment approaches for ICU patients at risk of vancomycin-related kidney injury by calculating the effects of vancomycin individually. By identifying pivotal risk factors and predicting the necessity for CKRT, our model could support physicians in developing tailored patient management strategies, especially when deciding on empirical vancomycin administration. This aligns with the goals of precision medicine to customize treatment plans based on the unique characteristics and risk profiles of individual patients (21).

This study comes with several limitations. The data set originates from a single center, and the number of patients included may not be sufficiently large for comprehensive machine learning and deep learning analyses. While causal inference analysis was conducted to assess the treatment effect of vancomycin, in practice, the choice of administering vancomycin is often dictated by the identified pathogen, leaving little to no alternative in many cases. It is important to note that causal inference analysis cannot substitute for Randomized Controlled Trials. For precise comparison and to establish causality definitively, randomized controlled trials are necessary. However, this investigation paves the way for future research, including the application of our methodology to different clinical data sets, the examination of more predictive variables, and the enhancement of machine learning models to improve predictive accuracy. This study lacks detailed socioeconomic status information for patients. Since ICU care is costly, the cohort may consist primarily of patients with higher socioeconomic status, introducing possible selection bias. Another limitation of this study is the relatively small number of patients requiring CKRT, comprising 3.1% of the total (41 patients). However, given that deep learning generally achieves higher accuracy with sparse outcomes compared to conventional statistical methods, the use of a deep learning-based causal inference model may partially mitigate this limitation (22, 23). Additionally, this study is the absence of therapeutic monitoring data, specifically serum vancomycin levels. Due to the limited number of patients with recorded serum levels, analyzing this subgroup could lead to reduced statistical power. Also, this study used US-based MIMIC-III data, and race was not included in the analysis. Validation in other race populations, including Asian patients, is needed to confirm the model’s applicability across diverse groups.

In conclusion, our study contributes to the expanding field of machine learning in critical care, providing significant insights into the predictors of CKRT application in blood culture positive patients and identifying the characteristics of patient groups more significantly affected by vancomycin.

## Data Availability

The data sets analyzed in the present study are publicly available and can be accessed through the MIMIC-III database from PhysioNet. This data set is publicly available through PhysioNet and provide the correct URL: https://physionet.org/content/mimiciii/1.4/.

## SUPPLEMENTAL MATERIAL

Supplemental materials spectrum.02662-24-s0001.docx Tables S1 to S4; Fig. S1 and S2.

ASM does not own the copyrights to Supplemental Material that may be linked to, or accessed through, an article. The authors have granted ASM a non-exclusive, world-wide license to publish the Supplemental Material files. Please contact the corresponding author directly for reuse.
