# Supplementary material for: Utilizing deep learning-based causal inference to explore vancomycin’s impact on continuous kidney replacement therapy necessity in blood culture-positive intensive care unit patients
Source: Microbiol Spectr. 2024 Dec 10;13(1):e02662-24. doi: 10.1128/spectrum.02662-24 (PMC11705918; doi:10.1128/spectrum.02662-24)

**Utilizing Deep Learning-based Causal Inference to Explore Vancomycin's Impact on Continuous Kidney Replacement Therapy Necessity in Blood Culture Positive Intensive Care Unit Patients**

Min Woo Kang, Yoonjin Kang

Supplementary Tables S1–S4

Supplementary Figures S1–S2

Supplementary Table S1. Baseline characteristics of train and tests dataset

|  | **Total**  **(N=1,318)** | **Train**  **(N=1,054)** | **Test**  **(N=264)** | **P-value^*^** |
| --- | --- | --- | --- | --- |
| **Age (years)** | 64.88 ± 15.0 | 64.85 ± 15.13 | 64.98 ± 14.5 | 0.896 |
| **Male (%)** | 764 (58.0) | 593 (56.3) | 171 (64.8) | 0.015 |
| **SBP (mmHg)** | 115.0 ± 25.36 | 115.53 ± 25.83 | 112.91 ± 23.23 | 0.111 |
| **DBP (mmHg)** | 64.19 ± 143.17 | 65.33 ± 159.85 | 59.65 ± 17.02 | 0.260 |
| **Heart rate (/min)** | 100.12 ± 21.55 | 100.19 ± 21.63 | 99.87 ± 21.23 | 0.827 |
| **SpO_2_ (%)** | 96.12 ± 6.66 | 96.08 ± 6.74 | 96.25 ± 6.32 | 0.705 |
| **Creatinine (mg/dL)** | 2.12 ± 2.1 | 2.06 ± 2.04 | 2.35 ± 2.30 | 0.057 |
| **Baseline creatinine (mg/dL)** | 1.88 ± 1.93 | 1.83 ± 1.90 | 2.06 ± 2.06 | 0.107 |
| **Intubation (%)** | 118 (9.0) | 93 (8.8) | 25 (9.5) | 0.835 |
| **Pseudomonas (%)** | 25 (1.9) | 16 (1.5) | 9 (3.4) | 0.078 |
| **Candidemia (%)** | 39 (3.0) | 28 (2.7) | 11 (4.2) | 0.275 |
| **MRSA (%)** | 132 (10.0) | 100 (9.5) | 32 (12.1) | 0.246 |
| **MSSA (%)** | 99 (7.5) | 80 (7.6) | 19 (7.2) | 0.931 |
| **pH** | 7.35 ± 0.12 | 7.35 ± 0.11 | 7.35 ± 0.12 | 0.863 |
| **Anion gap (mmol/L)** | 16.79 ± 5.17 | 16.76 ± 5.06 | 16.9 ± 5.56 | 0.720 |
| **Bicarbonate (mmol/L)** | 21.81 ± 6.34 | 21.81 ± 6.35 | 21.78 ± 6.33 | 0.933 |
| **WBC (10³/μL)** | 13.89 ± 11.44 | 13.84 ± 12.04 | 14.10 ± 8.63 | 0.680 |
| **Hemoglobin (g/dL)** | 10.56 ± 2.11 | 10.59 ± 2.12 | 10.42 ± 2.03 | 0.247 |
| **Platelet (10³/μL)** | 223.34 ± 137.59 | 222.24 ± 138.07 | 227.75 ± 135.60 | 0.557 |
| **aPTT (sec)** | 40.1 ± 24.34 | 39.88 ± 24.43 | 40.98 ± 23.99 | 0.507 |
| **PT INR** | 1.77 ± 1.38 | 1.77 ± 1.42 | 1.77 ± 1.17 | 0.981 |
| **Sodium (mmol/L)** | 138.08 ± 5.86 | 138.06 ± 5.85 | 138.19 ± 5.90 | 0.748 |
| **Potassium (mmol/L)** | 4.19 ± 0.82 | 4.19 ± 0.83 | 4.18 ± 0.81 | 0.828 |
| **Norepinephrine (mcg/kg/min)** | 0.19 ± 0.97 | 0.20 ± 1.01 | 0.17 ± 0.82 | 0.654 |
| **Vancomycin (%)** | 156 (11.8) | 122 (11.6) | 34 (12.9) | 0.631 |

Abbreviation: CRRT, continuous renal replacement therapy; SBP, systolic blood pressure; DBP, diastolic blood pressure; SpO_2_, oxygen saturation; MRSA, Methicillin-resistant Staphylococcus aureus; MSSA, Methicillin-susceptible Staphylococcus aureus; WBC, white blood cell; aPTT, activated partial thromboplastin time; PT INR, prothrombin time international normalized ratio

^*^Chi-square test for categorical variables and T-test for continuous variables

Supplementary Table S2. Odds ratio for continuous kidney replacement therapy application

|  | **Odds ratio (95% CI)** | **P-value** |
| --- | --- | --- |
| **Age** | 0.97 (0.94-1.00) | 0.023 |
| **Male** | 0.53 (0.25-1.10) | 0.086 |
| **Systolic blood pressure** | 1.00 (0.98-1.02) | 0.908 |
| **Diastolic blood pressure** | 0.98 (0.96-1.01) | 0.263 |
| **Heart Rate** | 1.01 (0.99-1.03) | 0.249 |
| **SpO_2_** | 1.00 (0.95-1.06) | 0.976 |
| **Creatinine** | 1.25 (0.82-1.90) | 0.295 |
| **Baseline Creatinine** | 1.01 (0.66-1.55) | 0.970 |
| **Intubation** | 10.25 (4.64-22.64) | <0.001 |
| **Vancomycin** | 2.63 (1.14-6.07) | 0.024 |

Adjusted variables: Pseudomonas, Candidemia, MSSA, MRSA, pH, anion gap, WBC, hemoglobin, platelet, aPTT, PT INR, sodium, potassium, norepinephrine

Abbreviation: CRRT, continuous renal replacement therapy; CI, confidence interval; SBP, systolic blood pressure; DBP, diastolic blood pressure; SpO_2_, oxygen saturation; MSSA, Methicillin-susceptible Staphylococcus aureus; MRSA, Methicillin-resistant Staphylococcus aureus; WBC, white blood cell; aPTT, activated partial thromboplastin time; PT INR, prothrombin time international normalized ratio

Supplementary Table S3. Comparing characteristics of two data groups with over and below predicted effect for continuous kidney replacement therapy initiation than median value when vancomycin administration in total dataset

|  | **Effect for CKRT < median value^*^** | **Effect for CKRT ≥ median value^*^** | **P-value^†^** |
| --- | --- | --- | --- |
| **Age (years)** | 69.00 ± 0.57 | 60.80 ± 0.55 | <0.001 |
| **Male (%)** | 362.0 (55.3) | 402.0 (60.6) | 0.140 |
| **SBP (mmHg)** | 119.14 ± 1.08 | 110.92 ± 0.87 | <0.001 |
| **DBP (mmHg)** | 70.69 ± 7.92 | 57.77 ± 0.61 | 0.104 |
| **Heart rate (/min)** | 93.61 ± 0.83 | 106.56 ± 0.77 | <0.001 |
| **SpO_2_ (%)** | 96.50 ± 0.26 | 95.74 ± 0.26 | 0.039 |
| **Creatinine (mg/dL)** | 1.74 ± 0.07 | 2.49 ± 0.09 | <0.001 |
| **Baseline creatinine (mg/dL)** | 1.53 ± 0.06 | 2.22 ± 0.08 | <0.001 |
| **Intubation (%)** | 53.0 (8.1) | 65.0 (9.8) | 0.333 |
| **Pseudomonas (%)** | 9.0 (1.4) | 16.0 (2.4) | 0.240 |
| **Candidemia (%)** | 18.0 (2.8) | 21.0 (3.2) | 0.776 |
| **MRSA (%)** | 61.0 (9.3) | 71.0 (10.7) | 0.464 |
| **MSSA (%)** | 41.0 (6.3) | 58.0 (8.8) | 0.115 |
| **pH** | 7.35 ± 0.00 | 7.34 ± 0.00 | 0.014 |
| **Anion gap (mmol/L)** | 15.86 ± 0.19 | 17.71 ± 0.21 | <0.001 |
| **Bicarbonate (mmol/L)** | 23.57 ± 0.25 | 20.06 ± 0.22 | <0.001 |
| **WBC (10³/μL)** | 14.95 ± 0.53 | 12.85 ± 0.35 | 0.001 |
| **Hemoglobin (g/dL)** | 10.58 ± 0.08 | 10.53 ± 0.08 | 0.655 |
| **Platelet (10³/μL)** | 284.50 ± 5.81 | 162.93 ± 3.62 | <0.001 |
| **aPTT (sec)** | 36.79 ± 0.89 | 43.37 ± 0.78 | <0.001 |
| **PT INR** | 1.64 ± 0.06 | 1.90 ± 0.05 | <0.001 |
| **Sodium (mmol/L)** | 138.54 ± 0.22 | 137.63 ± 0.23 | 0.005 |
| **Potassium (mmol/L)** | 4.17 ± 0.03 | 4.21 ± 0.03 | 0.404 |
| **Norepinephrine (mcg/kg/min)** | 0.17 ± 0.03 | 0.22 ± 0.04 | 0.331 |

Abbreviation: CKRT, continuous kidney replacement therapy; SBP, systolic blood pressure; DBP, diastolic blood pressure; SpO_2_, oxygen saturation; MRSA, Methicillin-resistant Staphylococcus aureus; MSSA, Methicillin-susceptible Staphylococcus aureus; WBC, white blood cell; aPTT, activated partial thromboplastin time; PT INR, prothrombin time international normalized ratio

**^*^**Median value: 0.074

**^†^**Chi-square test for categorical variables and T-test for continuous variables

Supplementary Table S4. Comparing characteristics of two data groups with over and below predicted effect for continuous kidney replacement therapy initiation than median value when vancomycin administration in train and test dataset

|  | **Train data** | | | **Test data** | | |
| --- | --- | --- | --- | --- | --- | --- |
|  | **Effect for CKRT < median value^*^** | **Effect for CKRT ≥ median value^*^** | **P-value^†^** | **Effect for CKRT < median value^*^** | **Effect for CKRT ≥ median value^*^** | **P-value^†^** |
| **Age (years)** | 69.01 ± 0.63 | 60.76 ± 0.64 | <0.001 | 68.94 ± 1.31 | 60.96 ± 1.14 | <0.001 |
| **Male (%)** | 273.0 (52.3) | 320.0 (60.2) | 0.050 | 89.0 (67.0) | 82.0 (62.6) | 0.662 |
| **SBP (mmHg)** | 120.14 ± 1.25 | 111.00 ± 0.96 | <0.001 | 115.22 ± 2.05 | 110.56 ± 2.03 | 0.104 |
| **DBP (mmHg)** | 72.91 ± 9.94 | 57.89 ± 0.70 | 0.104 | 61.97 ± 1.65 | 57.30 ± 1.30 | 0.025 |
| **Heart rate (/min)** | 93.74 ± 0.93 | 106.52 ± 0.87 | <0.001 | 93.08 ± 1.89 | 106.76 ± 1.64 | <0.001 |
| **SpO_2_ (%)** | 96.47 ± 0.29 | 95.71 ± 0.30 | 0.039 | 96.62 ± 0.61 | 95.87 ± 0.50 | 0.334 |
| **Creatinine (mg/dL)** | 1.74 ± 0.08 | 2.36 ± 0.10 | <0.001 | 1.71 ± 0.14 | 3.00 ± 0.24 | <0.001 |
| **Baseline creatinine (mg/dL)** | 1.54 ± 0.07 | 2.12 ± 0.09 | <0.001 | 1.50 ± 0.13 | 2.62 ± 0.21 | <0.001 |
| **Intubation (%)** | 43.0 (8.2) | 50.0 (9.4) | 0.587 | 10.0 (7.5) | 15.0 (11.5) | 0.391 |
| **Pseudomonas (%)** | 5.0 (1.0) | 11.0 (2.1) | 0.224 | 4.0 (3.0) | 5.0 (3.8) | 0.982 |
| **Candidemia (%)** | 15.0 (2.9) | 13.0 (2.4) | 0.81 | 3.0 (2.3) | 8.0 (6.1) | 0.213 |
| **MRSA (%)** | 47.0 (9.1) | 53.0 (10.0) | 0.678 | 14.0 (10.5) | 18.0 (13.7) | 0.554 |
| **MSSA (%)** | 31.0 (5.9) | 49.0 (9.2) | 0.064 | 10.0 (7.5) | 9.0 (6.9) | 1 |
| **pH** | 7.35 ± 0.01 | 7.34 ± 0.01 | 0.014 | 7.36 ± 0.01 | 7.33 ± 0.01 | 0.014 |
| **Anion gap (mmol/L)** | 16.02 ± 0.21 | 17.49 ± 0.23 | <0.001 | 15.24 ± 0.43 | 18.58 ± 0.51 | <0.001 |
| **Bicarbonate (mmol/L)** | 23.57 ± 0.28 | 20.09 ± 0.25 | <0.001 | 23.57 ± 0.56 | 19.95 ± 0.51 | <0.001 |
| **WBC (10³/μL)** | 15.25 ± 0.64 | 12.45 ± 0.38 | 0.001 | 13.77 ± 0.71 | 14.44 ± 0.81 | 0.529 |
| **Hemoglobin (g/dL)** | 10.58 ± 0.09 | 10.60 ± 0.09 | 0.655 | 10.59 ± 0.18 | 10.26 ± 0.18 | 0.187 |
| **Platelet (10³/μL)** | 289.54 ± 6.40 | 156.21 ± 3.93 | <0.001 | 264.73 ± 13.73 | 190.21 ± 8.60 | <0.001 |
| **aPTT (sec)** | 36.43 ± 1.00 | 43.26 ± 1.11 | <0.001 | 38.17 ± 2.00 | 43.83 ± 2.20 | 0.056 |
| **PT INR** | 1.65 ± 0.07 | 1.89 ± 0.06 | <0.001 | 1.59 ± 0.07 | 1.96 ± 0.12 | 0.012 |
| **Sodium (mmol/L)** | 138.58 ± 0.25 | 137.54 ± 0.26 | 0.005 | 138.38 ± 0.51 | 137.99 ± 0.54 | 0.599 |
| **Potassium (mmol/L)** | 4.19 ± 0.03 | 4.19 ± 0.04 | 0.404 | 4.08 ± 0.06 | 4.29 ± 0.08 | 0.035 |
| **Norepinephrine (mcg/kg/min)** | 0.18 ± 0.04 | 0.21 ± 0.05 | 0.331 | 0.10 ± 0.04 | 0.24 ± 0.09 | 0.170 |

Abbreviation: CKRT, continuous kidney replacement therapy; SBP, systolic blood pressure; DBP, diastolic blood pressure; SpO_2_, oxygen saturation; MRSA, Methicillin-resistant Staphylococcus aureus; MSSA, Methicillin-susceptible Staphylococcus aureus; WBC, white blood cell; aPTT, activated partial thromboplastin time; PT INR, prothrombin time international normalized ratio

**^*^**Median value: 0.074

**^†^**Chi-square test for categorical variables and T-test for continuous variables

Supplementary Figure S1. The receiver operating characteristic curves of deep learning-based causal inference models. A, Train dataset. B, Test dataset.

A


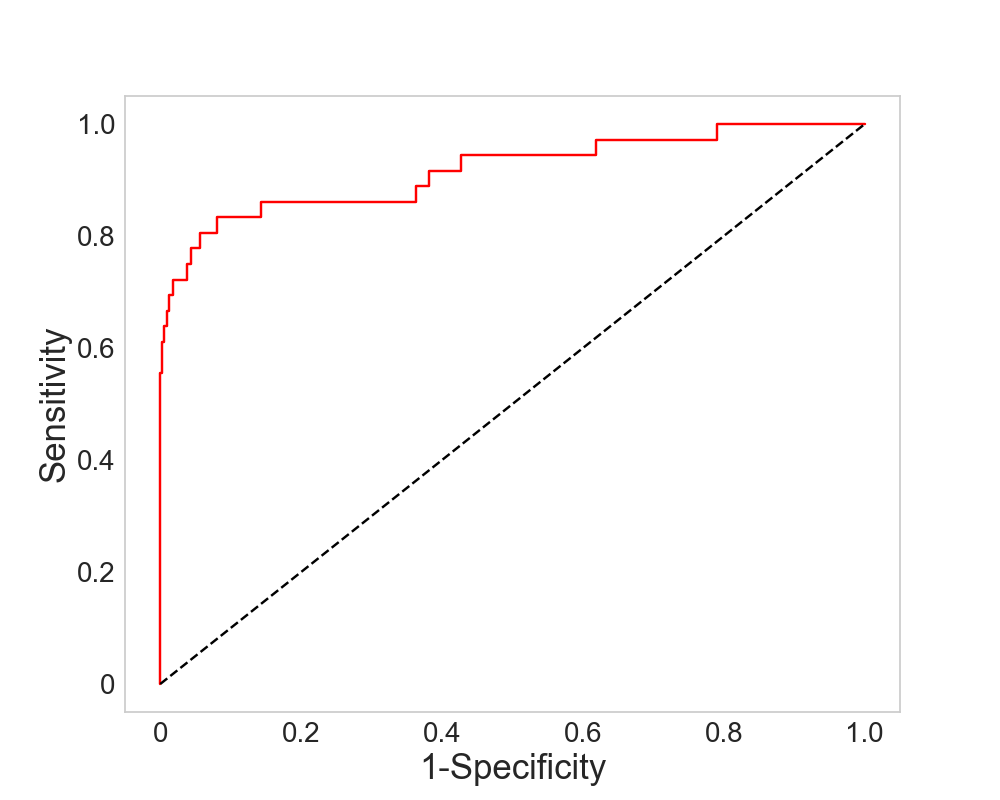


B


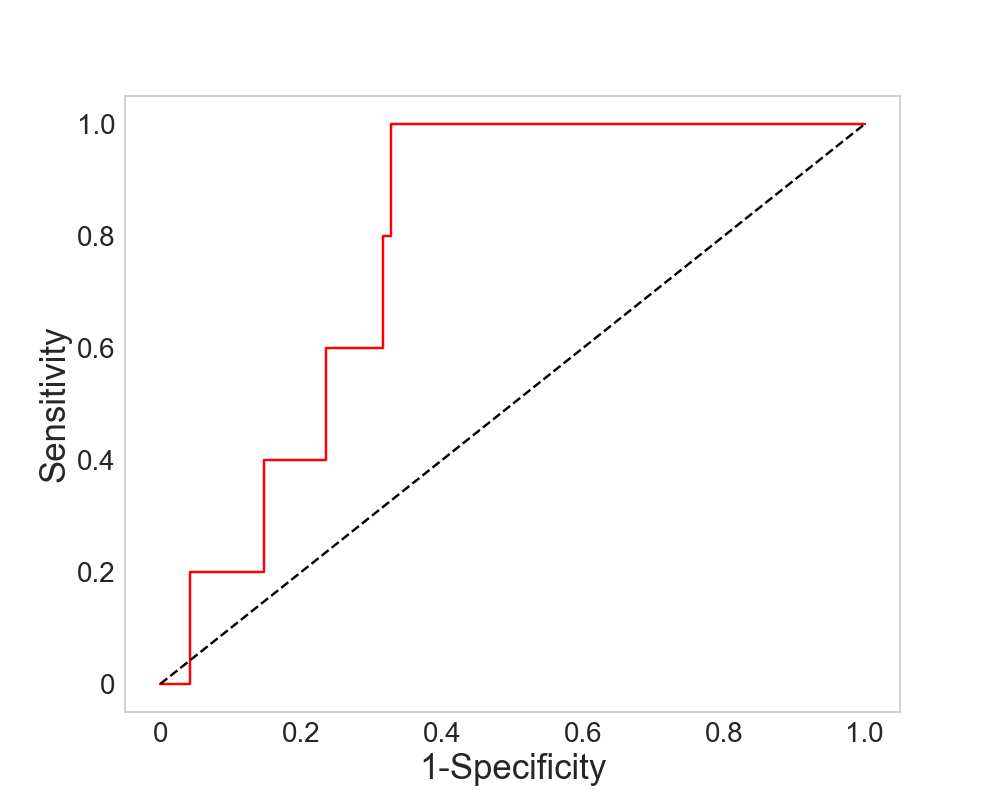


Supplementary Figure S2. Individual Treatment Effect Using Deep Learning-Based Causal Inference Model for Assessing Vancomycin Susceptibility in Individual Patients. A, Patients example 1. B, Patients example 2. SBP, systolic blood pressure; DBP, diastolic blood pressure; SpO2, oxygen saturation; MRSA, Methicillin-resistant Staphylococcus aureus; MSSA, Methicillin-susceptible Staphylococcus aureus; Cr, creatinine; WBC, white blood cell; Hb, hemoglobin; aPTT, activated partial thromboplastin time; PT INR, prothrombin time international normalized ratio; CKRT, continuous kidney replacement therapy.

A


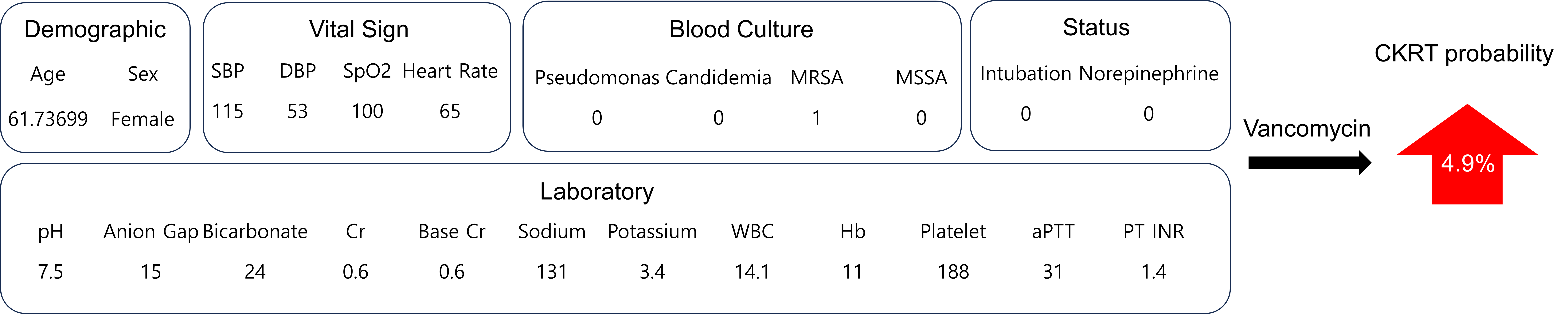


B


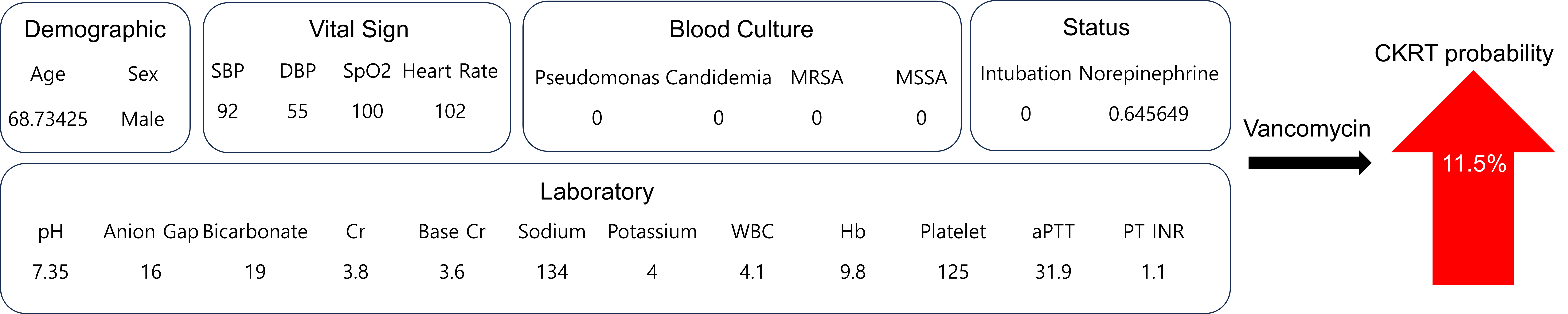

Supplement: Supplemental materials — Tables S1 to S4; Fig. S1 and S2. [file spectrum.02662-24-s0001.docx]
